# Genomic surveillance indicates clonal replacement of hypervirulent *Klebsiella pneumoniae* ST881 and ST29 lineage strains *in vivo*

**DOI:** 10.3389/fmicb.2024.1375624

**Published:** 2024-02-19

**Authors:** Ning Liu, Ningjie Lou, Jiajie Huang, Zhenhao Chen, Bing Li, Zhongheng Zhang, Yucai Hong, Liping Cao, Wei Xiao

**Affiliations:** ^1^Department of Emergency Medicine, Sir Run Run Shaw Hospital, Zhejiang University School of Medicine, Hangzhou, China; ^2^Department of Clinical Laboratory, Sir Run Run Shaw Hospital, Zhejiang University School of Medicine, Hangzhou, China; ^3^Department of General Surgery, Sir Run Run Shaw Hospital, Zhejiang University School of Medicine, Hangzhou, China

**Keywords:** *Klebsiella pneumoniae*, hypervirulent, whole genome sequencing, clonal replacement, infection episode

## Abstract

The emergence of hypervirulent *Klebsiella pneumoniae* (hvKp) poses a significant public health threat, particularly regarding its carriage in the healthy population. However, the genomic epidemiological characteristics and population dynamics of hvKp within a single patient across distinct infection episodes remain largely unknown. This study aimed to investigate the clonal replacement of hvKp K2-ST881 and K54-ST29 lineage strains in a single patient experiencing multiple-site infections during two independent episodes. Two strains, designated EDhvKp-1 and EDhvKp-2, were obtained from blood and cerebrospinal fluid during the first admission, and the strain isolated from blood on the second admission was named EDhvKp-3. Whole-genome sequencing, utilizing both short-read Illumina and long-read Oxford Nanopore platforms, was conducted. *In silico* multilocus sequence typing (MLST), identification of antimicrobial resistance and virulence genes, and the phylogenetic relationship between our strains and other *K. pneumoniae* ST881 and ST29 genomes retrieved from the public database were performed. Virulence potentials were assessed through a mouse lethality assay. Our study indicated that the strains were highly susceptible to multiple antimicrobial agents. Plasmid sequence analysis confirmed that both virulence plasmids, pEDhvKp-1 (166,008 bp) and pEDhvKp-3 (210,948 bp), belonged to IncFIB type. Multiple virulence genes, including *rmpA*, *rmpA2*, *rmpC*, *rmpD*, *iroBCDN*, *iucABCD*, and *iutA*, were identified. EDhvKp-1 and EDhvKp-2 showed the closest relationship to strain 502 (differing by 51 SNPs), while EDhvKp-3 exhibited 69 SNPs differences compared to strain TAKPN-1, which all recovered from Chinese patients in 2020. In the mouse infection experiment, both ST881 EDhvKp-1 and ST29 EDhvKp-3 displayed similar virulence traits, causing 90 and 100% of the mice to die within 72 h after intraperitoneal infection, respectively. Our study expands the spectrum of hvKp lineages and highlights genomic alterations associated with clonal switching between two distinct lineages of hvKP that successively replaced each other *in vivo*. The development of novel strategies for the surveillance, diagnosis, and treatment of high-risk hvKp is urgently needed.

## Introduction

*Klebsiella pneumoniae* is a prevalent nosocomial pathogen responsible for various infections, including pneumonia, urinary tract infection, liver abscess, bacteremia and sepsis (Paczosa and Mecsas, 2016). Although *K. pneumoniae* has numerous genetic and phenotypic diversities, its successful prevalence in clinical settings can be attributed to antimicrobial resistance and hypervirulence (Lai et al., 2019; Choby et al., 2020). In 1986, seven *K. pneumoniae* isolates were cultured from a series cases of pyogenic liver abscess complicated by septic endophthalmitis in China Taiwan (Liu et al., 1986). Afterwards, such kind of *K. pneumoniae* isolates that associated with life-threatening systemic or multiple sites infections were designated as hypervirulent *K. pneumoniae* (hvKp) to be distinguish from classical *K. pneumoniae* (cKp) (Russo and Marr, 2019). hvKp represents a distinct and concerning variant of the bacterium that has garnered increasing attention due to its heightened pathogenicity and ability to cause severe infections and poorer patient outcomes with notable clinical impact. hvKp strains exhibit the distinctive characteristic of expressing highly mucoid capsules, resulting in a “hypermucoviscous” colony phenotype (Shon et al., 2013). Their increased virulence is typically ascribed to the carriage of several large, non-conjugative “hypervirulence” plasmids by a few specific clonal lineages and capsule loci (Wyres et al., 2020). The emergence and global spread of hvKp strains pose a significant threat to public health, necessitating a comprehensive understanding of their molecular epidemiological characteristics, mechanisms of virulence, and associated clinical implications.

Though hvKp has been studied for nearly 40 years, its definition remains unclear and controversial (Chen et al., 2023). Many studies summarized the differences between hvKp and CKP, clinical manifestations, capsule typing, hypermucoviscosity, and virulence genes are helpful to identify hvKp (Choby et al., 2020). For example, hvKp isolates often have hypermucoviscous phenotype and carry plasmids encoding virulence genes, and are associated with community-acquired infections in immunocompetent hosts (Struve et al., 2015). Most importantly, hvKp isolates were found more virulent (Lethal dose <10^3^ CFU) than CKP (Lethal dose >10^6^ CFU) in mouse infection experiments (Russo and MacDonald, 2020; Russo et al., 2021). Notably, many studies have shown that sequence type (ST)23, ST65, ST86 and capsular type K1, K2 were closely associated with hvKp (Liao et al., 2014).

While the majority of hvKp strains exhibit susceptibility to various classes of antimicrobial agents, there have been reports of multidrug-resistant hvKp strains (DeLeo et al., 2023). The emergence of these strains poses significant therapeutic challenges, contributing to a higher morbidity and mortality rates. Addressing the complexities associated with the treatment of multidrug-resistant hvKp infections becomes paramount in mitigating the clinical impact of these formidable strains (Lam et al., 2021). Of particular concern is the emergence of hypervirulent carbapenem-resistant *K. pneumoniae*, capable of causing challenging-to-treat infections with adverse outcomes (Gorrie et al., 2022). Over the past decade, hv-CRKP has rapidly disseminated, often attributed to the acquisition of pLVPK-like virulence plasmids containing prevalent virulence genes, such as the mucoid regulator gene (*rmpA2*), distinct mucoid regulator operon (*rmpADC*), salmochelin receptor gene (*iroN*), salmochelin biosynthesis genes (*iroBCD*), aerobactin receptor gene (*iutA*), and aerobactin biosynthesis genes (*iucABCD*) (Heng et al., 2023; Liu et al., 2023). Our previous study also highlighted the global evolution and geographic diversity of hypervirulent carbapenem-resistant *K. pneumoniae* from 1980 to 2022 in 105 countries (Wu et al., 2022). Moreover, the capsule polysaccharide of *K. pneumoniae* is recognized as a crucial virulence factor, conferring resistance to phagocytosis and serum bactericidal activity (Opoku-Temeng et al., 2019). To date, over 100 capsular (K) serotypes of *K. pneumoniae* have been characterized. Significantly, several studies highlight the robust association of K1 and K2 serotypes with hypervirulent *K. pneumoniae* phenotype (Liao et al., 2014). However, it’s noteworthy that the K54 capsular type, especially within representatives of sequence type ST29, has also been linked to hypervirulence recently, expanding the spectrum of capsular serotypes implicated in this enhanced virulence phenotype (Shao et al., 2022).

Although previous studies have extensively explored the molecular epidemiology, virulence factors, and antimicrobial resistance of hvKp, the genomic epidemiological characteristics and population dynamics of hvKp within a single patient across distinct infection episodes remain largely unknown. In this study, we isolated a K2-ST881 and a K54-ST29 *K. pneumoniae* strain from a single patient suffering multiple site infection in two independent episodes. In addition, we performed a comprehensive genomic epidemiology analysis between hvKp isolates from this study and global ST881 and ST29 *K. pneumoniae* isolates retrieved from public database to enhance understanding of the potential evolutionary history of hvKp. Finally, the virulence potentials of both isolates were measured by mouse lethality assay. Our study highlights the genomic alterations correlating with clonal switch between two distinct lineages of hvKp that successively replaced each other *in vivo*.

## Materials and methods

### Bacterial strains

Three strains were recovered from the cerebrospinal fluid (CSF) and blood of a 32-year-old man who had been admitted twice to a tertiary hospital in Hangzhou, Zhejiang Province, China. In March 2021, the patient was admitted with fever, persistent headache and vomiting. Later in December 2021, he was admitted again due to chill and fever. The patient had a history of gastrointestinal hemorrhage and kept drinking alcohol in recent years. The isolate was identified *K. pneumoniae* by VITEK-2 (BioMérieux, France) and confirmed using MALDI-TOF mass spectrometry (Bruker, Germany). Two strains obtained from blood and CSF on the first admission were designated EDhvKp-1 and EDhvKp-2 respectively, the strain isolated from blood on the second admission was named EDhvKp-3.

### Antimicrobial susceptibility testing

Antimicrobial susceptibility testing was performed using VITEK-2 compact system (BioMérieux, France). *E. coli* ATCC 25922 and *K. pneumoniae* ATCC 700603 served as the quality controls. Antibiotics resistance breakpoints were interpreted according to the Clinical and Laboratory Standards Institute (CLSI) 2023 and European Committee on Antimicrobial Susceptibility Testing (EUCAST) v13 standards.

### Whole-genome sequencing and bioinformatics analysis

The genomic DNA of all the CRKP isolates was extracted by a QIAamp DNA Mini Kit (QIAGEN, Hilden, Germany), and the DNA concentration was quantified using Nanodrop (Thermo Scientific, Waltham, United States). Then, the libraries were prepared using the TruePrep™ DNA Library Prep Kit V2 (Vazyme, Nanjing, China). The genome of three isolates were sequenced using Illumina NovaSeq 6000 (Illumina, San Diego, CA, United States) and Oxford Nanopore GridION (Nanopore, Oxford, United Kingdom) platforms. The sequencing data of both short and long reads were assembled using Unicycler v0.5.0 (Wick et al., 2017). The genome annotation was conducted by the Prokaryotic Genome Annotation Pipeline (PGAP). The sequence types (ST), KL types, and virulence genes were analyzed by Kleborate and the BacWGSTdb 2.0 server (Feng et al., 2021; Lam et al., 2021). Antimicrobial resistance genes were analyzed by ABRicate with default parameters based on the National Center for Biotechnology Information (NCBI) AMRFinderPlus database. A circular comparison of different hypervirulence plasmids were created and illustrated using BLAST Ring Image Generator (BRIG) (Alikhan et al., 2011). The functional categorization and analysis of integrative and conjugative elements in bacteria were performed by ICEberg 3.0 (Wang et al., 2024).

### Single-nucleotide polymorphisms and phylogenetic analysis

To provide global context, 25,852 publicly available *K. pneumoniae* genomes were collected from the NCBI GenBank database on January 12, 2024. A total of 10 and 179 *K. pneumoniae* isolates were classified to ST881 and ST29, respectively. The assembled genome sequence data, comprising 19 complete and 170 draft genome sequences, was directly retrieved from the NCBI GenBank database. The raw reads were acquired from the NCBI SRA database and subsequently assembled locally using Unicycler v0.5.0 (Wick et al., 2017). The phylogenetic tree generated by cgSNP analysis was performed using Snippy v4.6.0. Strain EDhvKp-1 and EDhvKp-3 were used as a reference genome for the ST881 and ST29 *K. pneumoniae* strains, respectively. The SNPs between each pair of isolates in different groups were calculated by snp-dists v0.8.2, and the recombination events were calculated by Gubbins v3.3.2 based on the alignments (Croucher et al., 2015; Wu et al., 2023a). Fasttree v2.1.11 infers an approximately-maximum-likelihood phylogenetic tree from these non-recombinant SNPs. The visualization and annotation of phylogenetic trees, along with the identification of antimicrobial resistance genes, virulence genes, KL types, and strain metadata, were carried out using the Interactive Tree of Life (iTOL) V6 web server (Letunic and Bork, 2021).

### Mouse lethality assay

To assess the virulence of the three strains, pathogen-free 6-week-old female CD1 mice were employed. A sample population of 10 mice was utilized for each strain, with each mouse intraperitoneally injected with 0.1 mL of a bacterial suspension at a concentration of 10^4^ CFU in 0.9% NaCl. Mortality rates were monitored for a period of 5 days. Additionally, liver specimens from deceased mice were collected for histological observation using hematoxylin and eosin (HE) staining. The strains XWKP27 and XWKP12, isolated from patients in the intensive care unit (ICU) and investigated in our prior study, were used as controls (Liu et al., 2023). XWKP27, a classical ST11 carbapenem-resistant *K. pneumoniae* strain lacking any virulence plasmid and displaying non-hypermucoviscosity, was unable to cause mortality in mouse infection experiments even at a 10^7^ CFU inoculation. XWKP12, a typical ST23 hypervirulent *K. pneumoniae* strain, possessing a virulence plasmid and hypermucoviscosity, caused mortality in mouse infection experiments at a 10^3^ CFU inoculation.

## Results

### Clinical characteristics and outcomes of the patient

**Figure 1:**
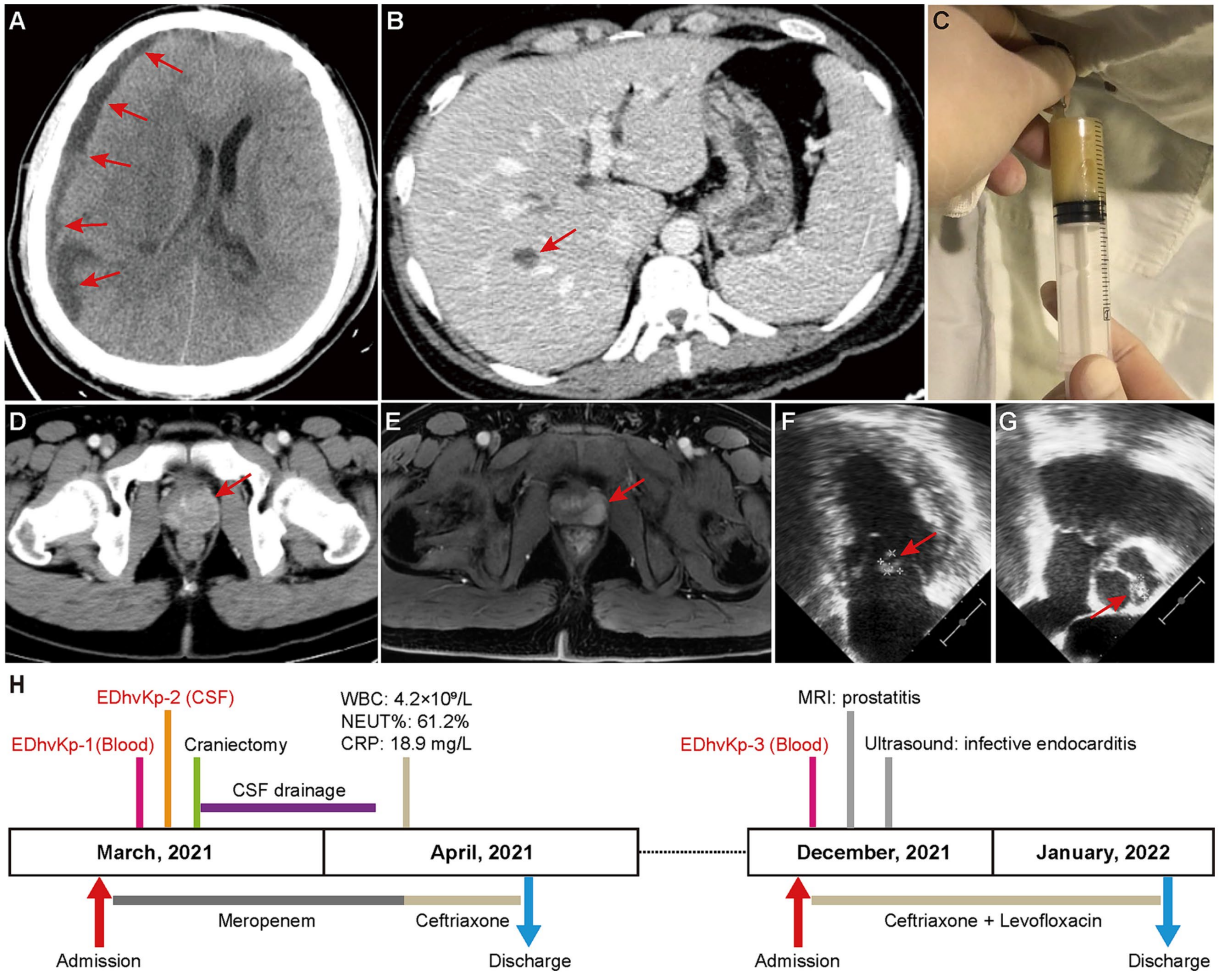
Clinical image findings and treatment process of this case. **(A)** CT-scan showed a subdural effusion compressing brain tissue. **(B)** CT-scan showed a liver abscess in right lobe, which disappeared in a subsequent CT scan after antibacterial treatment, supporting the diagnosis of a liver abscess. **(C)** Purulent CSF was drained by lumbar puncture. **(D,E)** Contrast-enhanced CT-scan and magnetic resonance imaging showed prostatitis. **(F,G)** Cardiac ultrasound suggested excrescences on the bicuspid valve (9.3 × 8.4 mm) and aortic valve (8.8 × 5.3 mm). **(H)** Clinical characteristics and treatment process of the patient.

On March 2021, the patient was admitted to emergency intensive care unit with fever (38.9°C), persistent headache, vomiting and unconsciousness. Physical examinations showed stiffness of the neck, laboratory tests suggested leukocytosis (27.2 × 10^9^/L), increased serum level of C-reactive protein (184.7 mg/L) and total bilirubin (44.6 μmol/L). CT-scan showed a subdural effusion compressing brain tissue (Figure 1A) and a liver abscess in right lobe (Figure 1B), lumbar puncture presented severe purulent CSF (Figure 1C), so meropenem was administrated empirically. Next generation sequencing of CSF reported *K. pneumoniae*, and β-lactams-susceptible *K. pneumoniae* were cultured from both CSF and blood. Shortly, the patient exhibited progressive disorder of consciousness and limb movement, hence decompressive craniectomy and CSF drainage were operated in emergency. Meropenem was continuously to be used to prevent other bacterial infection until the patient’s situation had been significantly improved. After that, ceftriaxone was used according to the antimicrobial de-escalation strategy. Finally, the patient recovered and was discharged a month later.

However, in December 2021, he was hospitalized again due to chill and fever for 1 day, with a body temperature up to 40°C, and blood culture isolated β-lactams-susceptible *K. pneumoniae* again. No obvious abnormality was seen in cranial CT-scan, but contrast-enhanced CT-scan and magnetic resonance imaging showed prostatitis (Figures 1D,E), and transesophageal echocardiography suggested excrescences on both bicuspid valve and aortic valve (Figures 1F,G), indicating infective endocarditis. Considering the difficulty of using ceftriaxone alone in eradicating the bacteria in both prostate and cardiac valve, the synergistic antibiotic combinations of ceftriaxone and levofloxacin was used to achieve a better clinical outcome. Fortunately, the infections were totally controlled after antibiotic therapy and the patient was discharged 3 weeks later. Follow-up of ultrasound at the 6^th^ month after discharge showed no excrescences on cardiac valves. The specific treatment process is shown in Figure 1H. Finally, the patient recovered completely without any obvious mental or physical disability.

### Antimicrobial susceptibility testing results

Three *K. pneumoniae* isolates were susceptible to most of the tested antibiotics, including aztreonam, cefepime, cefotetan, ceftazidime, ceftriaxone, ciprofloxacin, levofloxacin, gentamycin, ertapenem, imipenem, piperacillin-tazobactam, tobramycin, cefazolin, and trimethoprim-sulfamethoxazole. They were only resistant to ampicillin and nitrofurantoin (Supplementary Table S1).

### Genomic characterization of the three *Klebsiella pneumoniae* isolates

**Figure 2:**
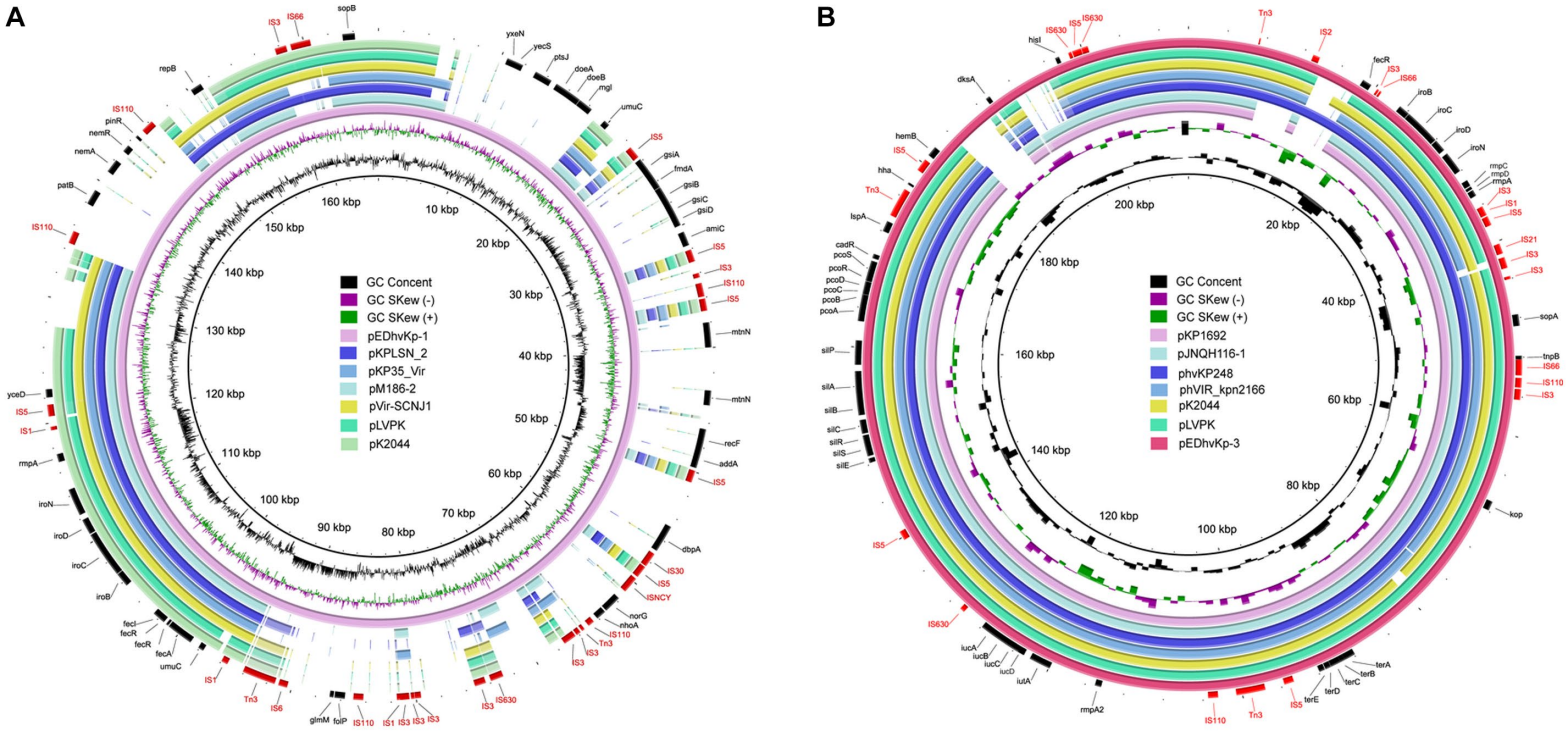
Genetic comparison of virulence plasmids, pEDhvKp-1 and pEDhvKp-3 recovered from EDhvKp-1 and EDhvKp-3 with similar plasmids in NCBI RefSeq database, respectively. **(A)** Alignment of similar virulence plasmids, pKPLSN_2 (CP13292), pKP35_Vir (CP132963), pM186-2 (CP063931), pVir-SCNJ1 (MK715436), pLVPK (AY378100), pK2044 (AP006726). pEDhvKp-1 were used as the reference plasmids. **(B)** Alignment of similar virulence plasmids, pKP1692 (CP041024), pJNQH116-1 (CP070899), phvKp248 (CP102439), phvVIR_kpn2166 (LR745046), pK2044 (AP006726), pLVPK (AY378100). pEDhvKp-3 was used as the reference plasmid.

Whole genome sequencing data revealed that EDhvKp-1 and EDhvKp-2 are originated from an identical clone, both strain contained a 5,315,853 bp chromosome and a 166,008 bp plasmid, named pEDhvKp-1. The chromosome carried several antimicrobial resistance genes, including *bla*_SHV-27_, *oqxB17*, *oqxA10*, and *fosA6*, conferring resistance to cephalosporin, phenicol, and fosfomycin, respectively. Virulence genes *rmpA*, *rmpC*, *rmpD*, *iroBCDN* and antimicrobial resistance gene *sul2* (sulfonamide resistance) all located on the plasmid pEDhvKp-1. The genome of strain EDhvKp-3 consisted of a 5,238,590 bp chromosome containing the antimicrobial resistance genes *oqxB25*, *oqxA10* and *fosA6*. Furthermore, multiple virulence genes *rmpA*, *rmpA2*, rmpC, rmpD, *iroBCDN*, *iucABCD* and *iutA* have been identified on the 210,948 bp plasmid pEDhvKp-3. However, the *rmpA2* genes were truncated by a loss-of-function mutation. Plasmid sequence analysis confirmed that both pEDhvKp-1 and pEDhvKp-3 belonged to IncFIB type. BLAST analysis of NCBI nucleotide database showed that the pEDhvKp-1 exhibited 46% query coverage and 99.79% identity with the pKPLSN_2 virulence plasmid (accession number CP132992) which harbored *iroBCDN* and *rmpA* (Figure 2A). The pEDhvKp-3 exhibited a high degree of nucleic acid similarity when compared to various virulence plasmids (Figure 2B), including phvVIR_kpn2166 (accession number LR745046, 93% query coverage, 99.84% nucleotide identity), pLVPK (accession number AY378100, 93% query coverage, 99.94% nucleotide identity) and pK2044 (accession AP006726, 93% query coverage, 99.39% nucleotide identity). Notably, these virulence plasmids all harbored multiple virulence genes, namely *rmpA*, *rmpA2*, *rmpC*, *rmpD*, *iroBCDN*, *iucABCD* and *iutA*. The functional categorization and analysis of integrative and conjugative elements revealed that ST881 and ST29 hypervirulent *K. pneumoniae* isolates lack any conjugative elements on the virulence plasmids.

### Phylogeny of global ST881 and ST29 isolates

**Figure 3:**
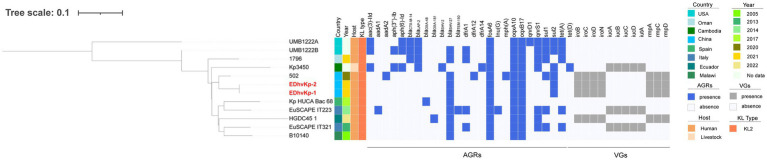
Phylogenetic analysis of EDhvKp-1, EDhvKp-2 and other 10 ST881 *Klebsiella pneumonia* strains retrieved from the NCBI GenBank database, strain EDhvKp-1 as reference. The strains identifier (ID), geographical location, separation time, hosts, KL typing, antimicrobial resistance genes and virulence-associated genes are shown. Diverse clusters are marked in different colors.

**Figure 4:**
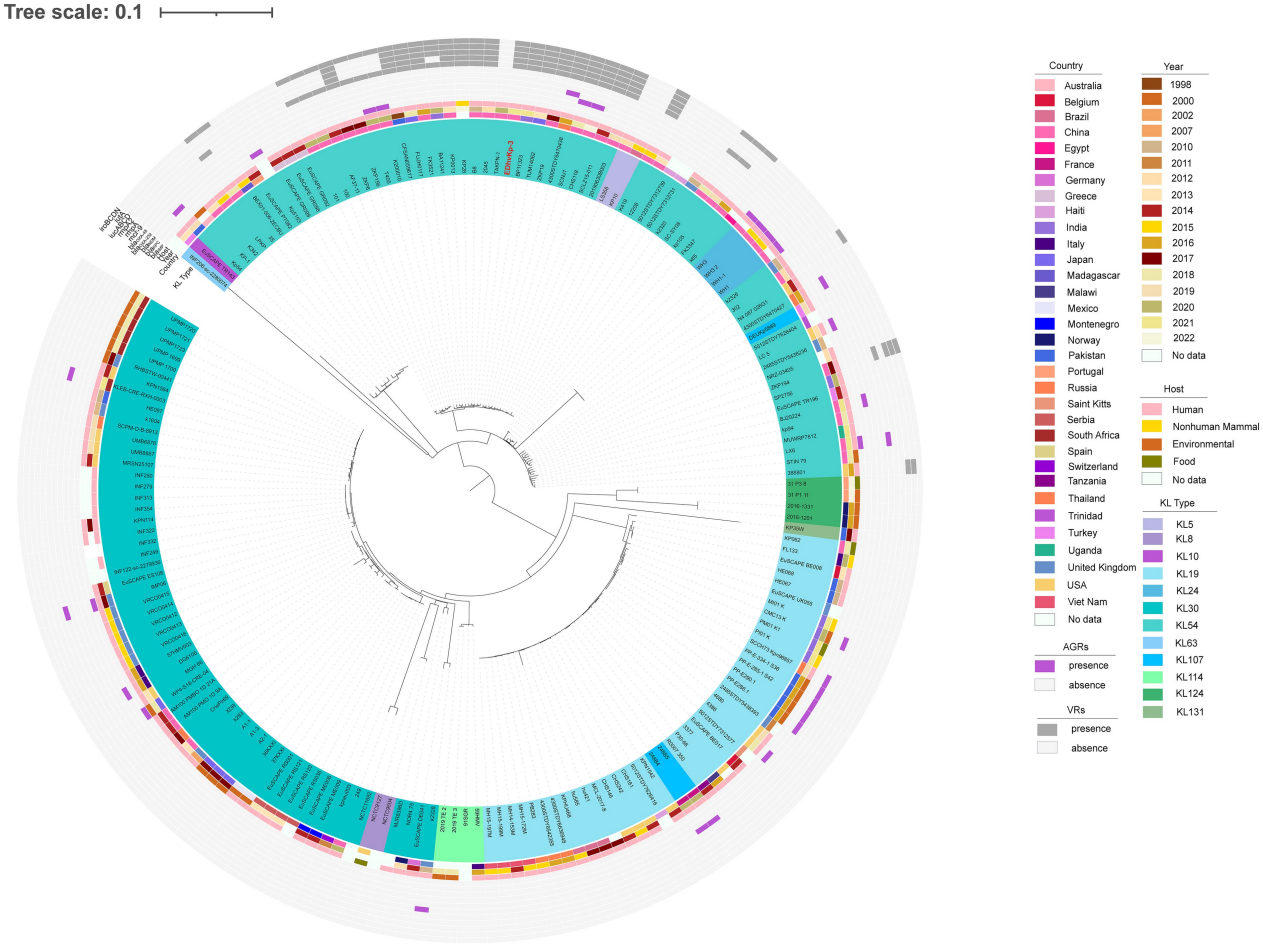
Phylogenetic analysis of EDhvKp-3 and other 179 ST29 *K. pneumonia* strains retrieved from the NCBI GenBank database, strain EDhvKp-3 as reference. The strains identifier (ID), geographical location, separation time, hosts, KL typing, carbapenemase genes, colistin resistance genes and virulence-associated genes are shown. Diverse clusters are marked in different colors.

MLST analysis showed that strain EDhvKp-1 and strain EDhvkp-2 belonged to ST881, while strain EDhvKp-3 belongs to ST29. Phylogenetic analysis of EDhvKp-1, EDHv-kp-2 and other 10 ST881 *K. pneumoniae* strains retrieved from the NCBI GenBank database suggested that the SNPs differences ranged from 0 to 385. The closest relative of EDhvKp-1 and EDhvKp-2 was strain 502 (differing by 51 SNPs), which recovered from the specimen of an infection patient in China Beijing in 2020 (Figure 3). Antimicrobial resistance genes fosfomycin (*fosA*) and quinolone (*oqxA10*, *oqxB17*) were observed in all ST881 *K. pneumoniae* strains. Additionally, the majority of the strains carried ESBLs [*bla*_CTX-M-14_ (25%), *bla*_SHV-2_ (8.3%) and *bla*_SHV-27_ (83.3%)] and other β-lactamases [*bla*_OXA-181_ (8.3%), *bla*_OXA-48_ (8.3%), *bla*_LAP-2_ (33.3%) and *bla*_TEM-150_ (8.3%)]. All ST881 *K. pneumoniae* strains were classified as KL2, with 4 strains (EDhvKp-1, EDhvKp-2, 502 and HGDC45_1) carrying the same virulence genes, namely *iroBCDN*, *rmpA*, *rmpC* and *rmpD*, and three strains (EuSCAPE_IT223, EuSCAPE_IT321 and Kp3450) carrying the *iucABCD* and *iutA* genes. In addition, cgSNP-based phylogenetic analysis of the ST29 lineage including EDhvKp-3 in this study suggested that 4 strains (2045, B8, KP28, TAKPN-1) exhibited close relationships with EDhvKp-3 (differing by <100 SNPs). Furthermore, 69 SNPs differences were identified between EDhvKp-3 and strain TAKPN-1, the latter strain have been isolated from the blood sample of a patient in Shandong in 2020 but lacking these virulence genes (Figure 4). The highest prevalence of ST29 *K. pneumoniae* was seen in China (41, 22.7%), followed by United States (16, 8.8%), United Kingdom (14, 7.7%), Pakistan (10, 5.5%) and Australia (9, 5.0%). The majority of strains (71.6%) were isolated from humans, while 12.2, 2.2 and 2.2% of strains were isolated from environment, food and animals, respectively. KL54 (62, 34.4%) was the most frequent KL type, followed by KL30 (57, 31.6%) and KL19 (39, 21.6%). 32 ST29 *K. pneumonia* harbored various virulence genes, with 18 strains simultaneously harboring *rmpA*, *rmpA2*, *rmpC*, *rmpD*, *iroBCDN*, *iucABCD* and *iutA* genes. Strain 4300STDY6470438, SCNJ1, CHS118, T405 and K200010 co-harbored virulence genes and carbapenem resistance genes (*bla*_OXA-232_, *bla*_NDM-5_, *bla*_NDM-1_, and *bla*_IMP-4_ respectively).

### Virulence traits of ST881 and ST29 isolates

**Figure 5:**
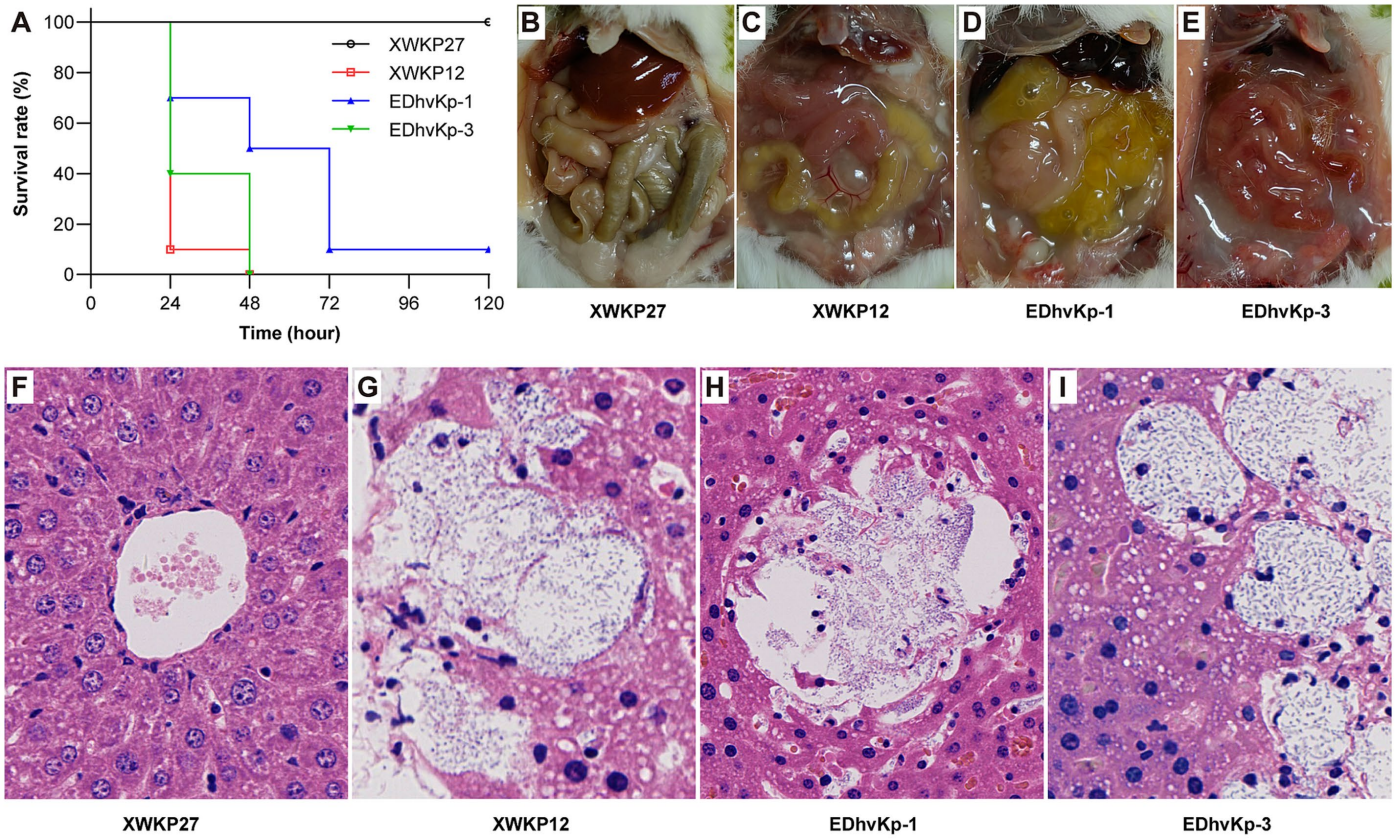
Results of mouse infection experiments. **(A)** The survival rate of mouse infected by strain EDhvKp-1 and EDhvKp-3. **(B–E)** The representative abdominal situation of infected mouse, severe intraperitoneal abscess and intestinal edema were observed in EDhvKp-1 and EDhvKp-3 group. **(F–I)** The representative histological changes of mouse livers under HE stains, abscess formation and bacterial proliferation were observed in EDhvKp-1 and EDhvKp-3 group. *K. pneumoniae* stain ST11 XWKP27 and ST23 XWKP12 served as the classical and hypervirulent control, respectively.

In the mouse infection experiment, all mice died after infection of hypervirulent ST23 XWKP12, the ST881 EDhvKp-1 and ST29 EDhvKp-3 showed similar virulence compared with XWKP12. 100, 90 and 100% of the mice died within 72 h after intraperitoneal infection of strain XWKP12, EDhvKp-1 and EDhvKp-3, respectively (Figure 5A), and they all suffered from severe intraperitoneal abscess and intestinal edema after intraperitoneal inoculation (Figures 5C–E). Since liver abscess is recognized as the most prominent and characteristic clinical manifestation of hvKp strains, we selected the liver as a representative organ that likely to develop abscesses due to the metastatic spread of strains, which aims to emulate the invasiveness of hvKp. HE stains of their livers suggested hepatocyte damage, abscess formation and bacterial proliferation (Figures 5G–I). Meanwhile, all mice survived in XWKP27 group and no obvious abnormality was observed in their abdominal cavity and livers (Figures 5B,F).

## Discussion

Hypervirulent *K. pneumoniae* (hvKp) has emerged as a significant global pathogen capable of causing both community-acquired and hospital-acquired infections, even in individuals without underlying health issues. This pathogen is harbored in the gastrointestinal tract, contributing to its dissemination in both community and healthcare settings (Russo and Marr, 2019). Initially identified in East Asia, hvKp gained prominence as a leading cause of pyogenic liver abscesses. Over subsequent decades, it has extended its reach globally, causing a spectrum of infections (Choby et al., 2020). An alarming trend is the emergence of multidrug-resistant hypervirulent strains, presenting a new and formidable challenge in the battle against this already perilous pathogen. Of particular concern is the convergence of virulence and carbapenem resistance (e.g., the *bla*_KPC-2_ gene carried by a pLVPK-like virulence plasmid) in a single *K. pneumoniae* hybrid plasmid, which is the most worrisome (Kong et al., 2021; Wu et al., 2023b).

HvKp is often found as part of the gut microbiome, contributing to its dissemination in both community and hospital settings. Previous study has identified *K. pneumoniae* in the stool of 10 to 35% of individuals (Choby et al., 2020). In hospitalized patients, the rates of *K. pneumoniae* intestinal colonization are notably higher, ranging between 19 and 38% (Martin et al., 2016; Gorrie et al., 2017). Furthermore, there is compelling evidence suggesting a direct link between gut colonization by hvKp and subsequent infection in the same individual. For instance, approximately half of ICU *K. pneumoniae* infections were found to be caused by the patient’s own gastrointestinal strain, emphasizing the role of gut colonization in the pathogenesis of hvKp infections (Gorrie et al., 2017). Another study also disclosed a moderate carriage rate of hvKp in the healthy population, ranging from 4 to 5.19% (Yang et al., 2022). There is a phylogenetically close relationship between intestinal colonization of hvKp and strains known to be implicated in clinical infections (Teo et al., 2024). These findings underscore the potential role of human gut as a reservoir for hvKp and suggest it may reside in the gut that contribute to infections in other body sites. In this case, the patient had a history of gastrointestinal hemorrhage and had kept drinking alcohol for several years. In the first episode, his headache started on the day after drinking and aggravated during the following week till admission. Hence, even though no *K. pneumoniae* strain was successfully isolated from fecal sample of the patient, may partially owing to antibiotic usage, it is reasonable to speculate that these life-threatening infections may result from translocations of intestinal colonized hvKp isolates under the circumstance of intestinal mucosal barrier impairment induced by alcohol. Therefore, active screening and antibiotic-mediated decolonization of *K. pneumoniae* from the gut of high-risk patients might be able to reduce the chance of hvKp infections.

The emergence of ST881 and ST29 hvKp strains reported in this study has presented unique challenges and implications for clinical management. The clinical implications of clonal replacement of hvKp strains underscore the adaptability and persistence of these pathogens within the host environment (Tang et al., 2023). This phenomenon may be influenced by a range of factors, including selective pressures, host immune responses, and the interplay between the bacterial strains and the host microenvironment. Taking the history of disease and drinking habit of this patient into consideration, his immune function of intestinal mucosal barrier was likely to be declined, leading to vulnerability to intestinal bacterial colonization and translocation, which may be responsible for his reinfection of different hvKp isolates.

Clinical examination of this patient suggested that multiple sites infections were developed in both episodes, indicating high virulence and pathogenicity of these two strains. In addition to intracranial and blood steam infection, the ST881 isolate EDhvKp-1 (EDhvKp-2) was also associated with a suspicious liver abscess, which disappeared on CT scan after antibiotic therapy. The ST29 isolate EDhvKp-3 caused blood steam infection, accompanied by prostatitis and infective cardiac endocarditis. Furthermore, the metastatically spreads of infection lesions were also observed in mouse lethality assay, while bacterial suspensions were inoculated into abdominal cavity, multiple micro abscesses were formed inside the livers. These findings were consistent with the clinical and microbiological features of hvKp reported previously. The virulence scoring system has been structured for evaluating the virulence level in *K. pneumoniae* assigned by a series of virulence-related factors (*ybt*, *clb* and *iuc*) in genome loci (Lam et al., 2021). In our study, the virulence score indicated that the ST881-KL2 isolate EDhvKp-1 was less virulent than the ST29-KL54 isolate EDhvKp-3. This result was consistent with our mouse lethality assay, suggesting that the complete virulence plasmid may enhance the virulence of isolate EDhvKp-3. Interestingly, the *rmpA2* gene was truncated in the EDhvKp-3, which suggested that the hypermucoviscosity in EDhvKp-3 was mainly mediated by other virulence genes rather than *rmpA2*.

Through cgSNP-based phylogenetic analysis, we found that 24 of 32 (75%) ST29-KL54 hvKp were originated from China, while the remaining were isolated from India (*n* = 2), Japan (*n* = 2), Madagascar (*n* = 1) and Thailand (*n* = 1). We hypothesized that the clonal transmission of ST29-KL54 occurred in China, which is consistent with the report of its dissemination in a tertiary hospital in China, 2018 (Qiu et al., 2023). A previous study reported a case of sepsis and brain abscess patient caused by an infection of the ST29-KL54 strain TAKPN-1 (Shao et al., 2022). This strain is most closely related to strain EDhvKp-3 in this study. However, strain TAKPN-1 lacks the pLVPK-like virulence plasmid, implying that the acquisition of the virulence genes by EDhvKp-3 may have occurred through horizontal transmission. EDhvKp-1 showed the highest degree of similarity to strain 502, which harbored extra virulence genes *iroBCDN*, *rmpA*, *rmpC* and *rmpD* and antimicrobial resistance genes *aadA1*, *dfrA12* and *mph* (A), in comparison to EDhvKp-1. We identified the KL2 in all *K. pneumoniae* lineages ST881, this particular serotype combination has been broadly associated with hvKp (Sanchez-Lopez et al., 2019). Of note, this was the first report of KL2-ST881 hvKp recovered from China.

We also confirmed the genetic contexts of *rmpA*, *rmpA2*, *rmpC*, *rmpD*, *iroBCDN*, *iucABCD* and *iutA* genes in pEDhvKp-3 plasmid shared high homology with pLVPK-like virulence plasmid. Diverse insertion sequences were discovered in the upstream and downstream regions of virulence genes, including *iroBCDN* with an upstream IS*3* and IS*66* transposase and *iucABCD* with an upstream IS*630* transposase, both of which were facilitated by horizontal transfer. Out of 180 ST29 *K. pneumoniae* strains, 30 (16.7%) were not of human origin. Previous studies have revealed the presence of hvKp in several sources including clinical settings, the environment and animals (Furlan et al., 2020; Mario et al., 2023). This presence contributed to a shared reservoir of antimicrobial resistance genes and virulence genes, posing a significant public health risk. Therefore, it is imperative to enhance the range of surveillance and implement the One Health strategy to prevent its further dissemination among different settings.

While this study represents the first investigation into the *in vivo* clonal replacement of two hypervirulent *K. pneumoniae* lineages ST881 and ST29, it is essential to acknowledge its limitations. A notable constraint is the relatively small sample size within a single patient, which may have an impact on the generalizability and robustness of the findings. In the future, it is imperative to conduct large-scale clinical studies involving patient cohorts and comprehensive investigations into the factors influencing clonal replacement of hvKp. This includes examining selection pressures, host immunological responses, and environmental factors, all of which are essential for advancing our understanding of hvKp infections. Furthermore, although the virulence traits of each strain were assessed through the identification of key virulence genes and animal lethality experiment, the origin and transmission dynamics of these hvKp strains are still unknown. Future research should not only concentrate on the genomic characteristics of hypervirulent strains but also delve into the gut colonization of hvKp directly precedes infection from the gut microbiome of health individuals.

## Conclusion

In conclusion, the clonal replacement of hypervirulent *K. pneumoniae* ST881 and ST29 lineages within the same patient emphasizes the complexity of hypervirulent *K. pneumoniae* infections, laying the groundwork for developing strategies to impede the persistence of these formidable bacterial pathogens. Our study provided a dynamic insight into clinical bacterial colonization and infection, highlighting the potential merit of microbiological screening in relatively healthy people, especially for those patients who have had systemic infection episodes or chronic impairments in their intestinal tracts. Further research into the genetic determinants, clinical outcomes, and factors influencing the dynamics of clonal replacement is essential for refining our understanding of the pathogenesis of hypervirulent *K. pneumoniae* and informing strategies for effective clinical management and infection control.

## Data availability statement

The genome sequences of the three *K. pneumoniae* isolates recovered in this study were deposited in the NCBI GenBank database under the BioProject accession number PRJNA553055.

## Ethics statement

The studies involving humans were approved by Ethics Committee of Sir Run Run Shaw Hospital, Zhejiang University School of Medicine, China. The human samples used in this study were acquired from a by-product of routine care. Written informed consent for participation was not required from the participants or the participants’ legal guardians/next of kin in accordance with the national legislation and institutional requirements. The animal study was approved by Ethics Committee of Sir Run Run Shaw Hospital, Zhejiang University School of Medicine, China. The study was conducted in accordance with the local legislation and institutional requirements.

## Author contributions

NLi: Data curation, Writing – original draft. NLo: Formal analysis, Investigation, Writing – original draft. JH: Data curation, Writing – original draft. ZC: Data curation, Writing – original draft. BL: Data curation, Writing – original draft. ZZ: Formal analysis, Visualization, Writing – review & editing. YH: Conceptualization, Project administration, Supervision, Validation, Writing – review & editing. LC: Project administration, Supervision, Writing – review & editing. WX: Conceptualization, Investigation, Methodology, Supervision, Visualization, Writing – original draft, Writing – review & editing.
