# Supplementary material for: Genomic surveillance indicates clonal replacement of hypervirulent Klebsiella pneumoniae ST881 and ST29 lineage strains in vivo
Source: Front Microbiol. 2024 Feb 19;15:1375624. doi: 10.3389/fmicb.2024.1375624 (PMC10910047; doi:10.3389/fmicb.2024.1375624)
Supplement: Supplementary file 1 [file Table_1.DOCX]

***Supplementary Material***

Supplementary Table S1. The minimum inhibitory concentrations for different antimicrobial agents of three *K. pneumoniae* isolates (μg/ml).

| Isolates | AMP | SAM | ATM | FEP | CTT | CAZ | CRO | CIP | LEV | GEN | ETP | IPM | NIT | TZP | TOB | CFZ | SXT |
| --- | --- | --- | --- | --- | --- | --- | --- | --- | --- | --- | --- | --- | --- | --- | --- | --- | --- |
| EDhvKp-1 | ≥32 | =8 | ≤1 | ≤1 | ≤4 | ≤1 | ≤1 | ≤0.25 | ≤0.25 | ≤1 | ≤0.5 | ≤1 | =128 | ≤4 | ≤1 | ≤4 | ≤20 |
| EDhvKp-2 | ≥32 | =4 | ≤1 | ≤1 | ≤4 | ≤1 | ≤1 | ≤0.25 | ≤0.25 | ≤1 | ≤0.25 | ≤1 | =128 | ≤4 | ≤1 | ≤4 | ≤20 |
| EDhvKp-3 | =16 | =4 | ≤1 | ≤1 | ≤4 | ≤1 | ≤1 | ≤0.25 | ≤0.25 | ≤1 | ≤0.5 | ≤1 | =64 | ≤4 | ≤1 | ≤4 | ≤20 |

AMP, Ampicillin; SAM, ampicillin-sulbactam; ATM, aztreonam; FEP, cefepime; CTT, cefotetan; CAZ, ceftazidime; CRO, ceftriaxone; CIP, ciprofloxacin; LEV, levofloxacin; GEN, gentamycin; ETP, ertapenem; IPM, imipenem; NIT, nitrofurantoin; TZP, piperacillin-tazobactam; TOB, tobramycin; CFZ, cefazolin; SXT, trimethoprim-sulfamethoxazole.
